# Supplementary material for: Heat adaptation of phage T7 under an extended genetic code
Source: Virus Evol. 2021 Dec 1;7(2):veab100. doi: 10.1093/ve/veab100 (PMC8923235; doi:10.1093/ve/veab100)
Supplement: veab100_Supp [file veab100_supp.zip › Sequencing_Analysis.zip!/Sequencing_Analysis/A1/evidence/JC_62.html]

BRESEQ :: Evidence


| New junction evidence | | | | | | | | | | | |
| --- | --- | --- | --- | --- | --- | --- | --- | --- | --- | --- | --- |
|  | | seq id | position | reads (cov) | reads (cov) | score | skew | freq | annotation | gene | product |
| \* | ? | NC\_001604.1 | = 13586 | 3 (0.000) | 2345 (0.770) | 347/592 | 0.3 | 93.9% | intergenic (–/–) | *–/–* | –/– |
|  | ? | NC\_001604.1 | 14356 = | 301 (0.100) |  |  |  |  | intergenic (–/–) | *–/–* | –/– |

Only 100 of 2393 total aligned reads displayed.  

`TTCCAGACGACTACGAGAAGTTTCGCTGGAGAGTCCCATTCTAATACGACTCACTAAAGGAGACACACCATGTTCAAACTGATTAAGAAGTTAGGCCAACTGCTGGTTCGTATGTACAACGTGGAAGCCAAGCGACTGAACGATGAGGCTCGTAAAGAGGCCACACAGTCACGCGCTCTGGCGATTCGCTCCAACGAACTGGCTGACAGTGCATCCACTAAAGTTACCGAGGCTGCCCGTGTGGCAAACCAAGCTCAACAGCTTTCCAAATTCTTTGAGTAATCAAACA‑GGAGAAACCATTATG‑‑‑‑‑‑‑‑‑‑‑‑‑‑‑‑‑‑‑‑‑‑‑‑‑‑‑‑‑‑‑‑‑‑‑‑‑‑‑‑‑‑‑‑‑‑‑‑‑‑‑‑‑‑‑‑‑‑‑‑‑‑‑‑‑‑‑‑‑‑‑‑‑‑‑‑‑‑‑‑‑‑‑‑‑‑‑‑‑‑‑‑‑‑‑‑‑‑‑‑‑‑‑‑‑‑‑‑‑‑‑‑‑‑‑‑‑‑‑‑‑‑‑‑‑‑‑‑‑‑‑‑‑‑‑‑‑‑‑‑‑‑‑‑‑‑‑‑‑‑‑‑‑‑‑‑‑‑‑‑‑‑‑‑‑‑‑‑‑‑‑‑‑‑‑‑‑‑‑‑‑‑‑‑‑‑‑‑‑‑‑‑‑‑‑‑‑‑‑‑‑‑‑‑‑‑‑‑‑‑‑‑‑‑‑‑‑‑‑‑‑‑‑‑‑‑‑‑‑‑‑‑‑‑‑‑‑‑‑‑‑‑‑‑‑‑‑‑‑‑‑‑‑‑‑‑‑‑‑‑‑‑‑‑‑‑‑‑‑‑‑‑‑‑‑‑‑‑‑‑‑‑‑‑‑‑‑‑‑‑‑‑‑‑‑‑‑‑‑‑  >  NC_001604.1/13283‑13586`  
`‑‑‑‑‑‑‑‑‑‑‑‑‑‑‑‑‑‑‑‑‑‑‑‑‑‑‑‑‑‑‑‑‑‑‑‑‑‑‑‑‑‑‑‑‑‑‑‑‑‑‑‑‑‑‑‑‑‑‑‑‑‑‑‑‑‑‑‑‑‑‑‑‑‑‑‑‑‑‑‑‑‑‑‑‑‑‑‑‑‑‑‑‑‑‑‑‑‑‑‑‑‑‑‑‑‑‑‑‑‑‑‑‑‑‑‑‑‑‑‑‑‑‑‑‑‑‑‑‑‑‑‑‑‑‑‑‑‑‑‑‑‑‑‑‑‑‑‑‑‑‑‑‑‑‑‑‑‑‑‑‑‑‑‑‑‑‑‑‑‑‑‑‑‑‑‑‑‑‑‑‑‑‑‑‑‑‑‑‑‑‑‑‑‑‑‑‑‑‑‑‑‑‑‑‑‑‑‑‑‑‑‑‑‑‑‑‑‑‑‑‑‑‑‑‑‑‑‑‑‑‑‑‑‑‑‑‑‑‑‑‑‑‑‑‑‑‑‑‑‑‑‑‑‑‑‑‑‑‑‑‑‑‑‑‑‑‑‑‑‑‑‑‑‑‑‑‑‑‑‑‑‑‑‑‑‑‑‑‑‑‑‑‑‑‑‑‑‑‑‑‑tatgATCGTTTCTGACATCGAAGCTAACGCCCTCTTAGAGAGCGTCACTAAGTTCCACTGCGGGGTTATCTACGACTACTCCACCGCTGAGTACGTAAGCTACCGTCCGAGTGACTTCGGTGCGTATCTGGATGCGCTGGAAGCCGAGGTTGCACGAGGCGGTCTTATTGTGTTCCACAACGGTCACAAGTATGACGTTCCTGCATTGACCAAACTGGCAAAGTTGCAATTGAACCGAGAGTTCCACCTTCCTCGTGAGAACTGTATTGACACCCTTGTGTTGTCACGTTTGATTCATTCCAAC  >  NC_001604.1/14356‑14655`  

                                                                                                                                                                                                                                                                                                                                                                                                                                                                                                                                                                                                                               
`TTCCAGACGACTACGAGAAGTTTCGCTGGAGAGTCCCATTCTAATACGACTCACTAAAGGAGACACACCATGTTCAAACTGATTAAGAAGTTAGGCCAACTGCTGGTTCGTATGTACAACGTGGAAGCCAAGCGACTGAACGATGAGGCTCGTAAAGAGGCCACACAGTCACGCGCTCTGGCGATTCGCTCCAACGAACTGGCTGACAGTGCATCCACTAAAGTTACCGAGGCTGCCCGTGTGGCAACCCAATCTCAACAACTTTCCAACTTCTTTTAGTAATCAAACA‑AGCAAAACCATT                                                                                                                                                                                                                                                                                                                 >  1:253710/1‑301`  
  `CCAGACGACTACGAGAAGTTTCGCTGGAGAGTCCCATTCTAATACGACTCACTAAAGGAGACACACCATGTTCAAACTGATTAAGAAGTTAGGCCAACTGCTGGTTCGTATGTACAACGTGGAAGCCAAGCGACTGAACGATGAGGCTCGTAAAGAGGCCACACAGTCACGCGCTCTGGCGATTCGCTCCAACGAACTGGCTGACAGTGCATCCACTAAAGTTACCGAGGCTGCCCGTGTGGCAAACCAAGCTCAACAGCTTTCCAAATTCTTTGAGTAATCAAACA‑GGAGAAATCAATAT                                                                                                                                                                                                                                                                                                               <  1:444402/301‑1`  
  `CCAGACGACTACGAGAAGTTTCGCTGGAGAGTCCCATTCTAATACGACTCACTAAAGGAGACACACCATGTTCAAACTGATTAAGAAGTTAGGCCAACTGCTGGTTCGTATGTACAACGTGGAAGCCAAGCGACTGAACGATGAGGCTCGTAAAGAGGCCACACAGTCACGCGCTCTGGCGATTCGCTCCAACGAACTGGCTGACAGTGCATCCACTAAAGTTACCGAGGCTGCCCGTGTGGCAAACCAAGCTCAACAGCTTTCCAAATTCTTTGAGTAATCAAACA‑GGAGAAACCATTAT                                                                                                                                                                                                                                                                                                               <  1:448470/301‑1`  
     `aACGACAACGAGAAGTTTCGCTGGAGAGTCACATTCTAATACGACTCACTAAAGGAGACACACCATGTTCAAACAGATTAAGAAATTAGGCCAACTGCTGGTTCGTATGTACAACGTGGAAGCCAAGCGACTGAACAATGAGGCTCGTAAAGAGGCCACACAGTCACGCGCTCTGGCGATTCGCTCCAACGAACTGGCTGACAGTGCATCCACTAAAGTTACCGAGGCTGCCCGTGTGGCAAACCAAGCTCAACAGCTTTCCAAATTCTTTGAGTAATCAAACA‑GGAGAAACCATTATGAT                                                                                                                                                                                                                                                                                                            <  1:85524/300‑1`  
                 `AAGTTTCGCTGGAGAGTCCCGTTCTAATACGACTCACTAAAGGATACACACCATGTTCAAACTGATTAAGAAGTTAGGCCAACTGCTGGTTCGTATGTACAACGTGGAAGCCAAGCGACTGAACGATGAGGCTCGGAAAGAGGCCACACAGTCACGCGCTCTGGCGATTCGCTCCAACGAACTGGCTGACAGTGCATCCACTAAAGTTACCGAGGCTGCCCGTGTGGAAAACCAAGCTCAACAGCTTTCCAAATTCTTTGAGTAATCAAACA‑GGAGAAACCATTATGATCGTATCTGACAT                                                                                                                                                                                                                                                                                                <  1:342939/301‑1`  
                      `TCGCTGGAGAGTCCCATTCTAATACGACTCACTAAAGGAGACACACCATGTTCAAACTGATTAAGAAGTTAGGCCAACTGCTGGTTCGTATGTACAACGTGGAAGCCAAGCGACTGAACGATGAGGCTCGTAAAGAGGCCACACAGTCACGCGCTCTGGCGATTCGCTCCAACGAACTGGCTGACAGTGCATCCACTAAAGTTACCGAGGCTGCCCGTGTGGCAAACAAAGCTCAACAGCTTTCCAAATTCTTTGAGTAATCAAACA‑GGAGAAACCATTATGATCGTTTCTGACATCGAAG                                                                                                                                                                                                                                                                                           >  1:359326/1‑301`  
                           `GGAGAGTCCCATTCTAATACGACTCACTAAAGGAGACACACCATGTTCAAACTGATTAAGAAGTTAGGCCAACTGCTGGTTCGTATGTACAACGTGGAAGCCAAGCGACTGAACGATGAGGCTCGTAAAGAGGCCACACAGTCACGCGCTCTGGCGATTCGCTCCAACGAACTGGCTGACCGTGCATCCACTAAAGTTACCGAGGCTGCCCGTGTGGCAAACCAAGCTCAACCGCTTTCCAAATTCTTTGATTAATCAAACA‑GGAGAAACCATTATGATCGTTTCTGACtgtctcttatac                                                                                                                                                                                                                                                                                      >  1:211355/1‑289`  
                                `GTCCCATTCTAATACGACTCACTAAAGGAGACACACCATGTTCAAACTGATTAAGAAGTTAGGCCAACTGCTGGTTCGTATGTACAACGTGGAAGCCAAGCGACTGAACGATGAGGCTCGTAAAGAGGCCACACAGTCACGCGCTCTGGCGATTCGCTCCAACGAACTGGCTGACAGTGCATCCACTAAAGTTACCGAGGCTGCCCGTGTGGCAAACCAAGCTCAACAGCTTTCCAAATTCTTTGAGTAATCAAACA‑GGAGAAATCAATATGATCGTTTCTGACATAGAAGCTAACGCCCT                                                                                                                                                                                                                                                                                 >  1:161463/1‑301`  
                                     `ATTCTAATACGACTCACTAAAGGAGACACACCATGTTCAAACTGATTAAGAAGTTAGGCCAACTGCTGGTTCGTATGTACAACGTGGAAGCCAAGCGACTGAACGATGAGGCTCGTAAAGAGGCCACACAGTCACGCGCTCTGGCGATTCGCTCCAACGAACTGGCTGACAGTGCATCCACTAAAGTTACCGAGGCTGCCCGTGTGGCAAACCAAGCTCAACAGCTTTCCAAATTCTTTGAGTAATCAAACA‑GGAGAAACCATTATGATCGTTTCTGACATCGAAGCTAACGCCCTCTTcg                                                                                                                                                                                                                                                                            >  1:356203/1‑299`  
                                      `TTCTAATACGACTCACTAAAGGAGACACACCATGTTCAAACTGATTAAGACGTTAGGCCAACTTATGGTTCGTATGTCCAACGTGGAAGCCAAGCGACTGAACGATAAGGCTCGTAAGGAGGCCCCACAGTCACGCGCTCTGGAGATTCGCTCCAACGCACTGGCAGCCAGTGCATCCACTAAAGTTACCGATGCTGCCCGTCTGGCACACCAAGCTCAACAGCTTTCCAAATTCATTGAGTAATCAAACA‑GTAGATATCAACATGATCGTTTCTGACATCGACGCTAACGCTCTCTTAaa                                                                                                                                                                                                                                                                           >  1:347254/1‑299`  
                                      `TTCTAATACGACTCACTAAAGGAGACACACCATGTTCAAACTGATTAAGAAGTTAGGCCAACTGCTGGTTCGTATGTACAACGTGGAAGCCAAGCGACTGAACGATGAGGCTCGTAAAGAGGCCACACAGTCACGCGCTCTGGCGATTCGCTCCAACGAACTGGCTGACAGTGCATCCACTAAAGTTACCGAGGCTGCCCGTGTGGCACACCAAGCTCAACAGCTTTCCAACTTCTTTGAGTAATCAAACA‑GGAGAAATCAATATGATCGTTTCTGACATCGAAGCTAACGCCATCTTAGA                                                                                                                                                                                                                                                                           >  1:204166/1‑301`  
                                      `TTCTAATACGACTCACTAAAGGAGACACACCATGTTCAAACTGATTAAGAAGTTAGGCCAACTGCTGGTTCGTATGTACAACGTGGAAGCCAAGCGACTGAACGATGAGGCTCGTAAAGAGGCCACACAGTCACGCGCTCTGGCGATTCGCTCCAACGAACTGGCTGACAGTGCATCCACTAAAGTTACCGAGGCTGCCCGTGTGGCAAACCAAGCTCAACAGCTTTCCAAATTCTTTGAGTAAT‑ACACACGGAGAAACCATTATGTTCGTTCATGACATCGAAGCctgtcacttatacac                                                                                                                                                                                                                                                                           >  1:174629/1‑286`  
                                      `TTCTAATACGACTCACTAAAGGAGACACACCATGTTCAAACTGATTAAGAAGTTAGGCCAACTGCTGGTTCGTATGTACAACGTGGAAGACAAGCGACTGAACGATGAGGCTCGTAAAGAGGCCACACAGTCACGCGCTCTGGCGATTCGCTCCAACGAACTGGCTGACAGTGCATCCACTAAAGTTACCGAGGCTGCCCGTGTGGCCAACCAAGCTCAACAGCTTTCCAAATTCTTTGAGTAATCAAACA‑GGAGAAACCATTATGATCGTTTCTGACATCGAAGCTAACGCCCTCTTAGA                                                                                                                                                                                                                                                                           >  1:62562/1‑301`  
                                       `ggtataagggacagaTAAATGAGACACACCATGTTCAAACAGATTAAGAAGTTAGGCCAACTGCTGGTTCGTATGTACAACGTGGAAGCCAAGCGACTGAACGATGAGACTCGTAAAGAGGCCACAAAGTAACGCGCTCTGGCGATTCGCTCCAACGAAATGACTGAAAGGGAATCCACTAAAGTTACCGAGGCTGCCCGAGTGGCAAACCAAGCTCAACAGCTTTCCAAATTCTTTGAGTAATCAAACA‑GGAGAAACCATTATGATCGTTTCTGACATCGAAGCTAACGCCCTCTTAGAG                                                                                                                                                                                                                                                                          <  1:373306/286‑1`  
                                       `TCTAATACGACTCACTACAGGCGACACACCATGTTCAAACTGATTAAGAAGTTAGGCCAACTGCTGGTTCGTATGTACAACGTGGAAGCCAAGCGACTGAACGATGAGGCTCGTAAAGAGGCCACACAGTCACGCGCTCTGGCGATTCGCTCCAACGAACTGGCTGACAGTGCATCCACTAAAGTTACCGAGGCTGCCCGTGTGGCAAACCAAGCTCAACAGCTTTCCAAATTCTTTGAGTAATCAAACA‑GGAGAAACCATTATGATCGTTTCTGACATCGAAGCTAACGCCCTCTTAGAG                                                                                                                                                                                                                                                                          <  1:428106/301‑1`  
                                       `TCTAATACGACTCACTAAAGGAGACACACCATGTTCAAACTGATTAAGAAGTTAGGCCAACTGCTGGTTCGTATGTACAACGTGGAAGCCAAGCGACTGAACGATGAGGCTCGTAAAGAGGCCACACAGTCACGCGCTCTGGCGATTCGCTCCAACGAACTGGCTGACAGTGCATCCACTAAAGTTACCGAGGCTGCCCGTGTGGCAAACCAAGCTCAACAGCTTTCCAAATTCTTTGAGTAATCAAACA‑GGAGAAATCAATATGATCGTTTCTGACATCGAAGCTAACGCCCTCTTAGAG                                                                                                                                                                                                                                                                          <  1:409063/301‑1`  
                                         `aAATACGACTCACTAAAGGAGACAAACCATGTTCAAACTGATGAAGAAGATAGGCCAACTGATGGTTCGTATGTACAACGTGGAAGCCAAGCGACTGAACTATGAGGCTCGTAAAGAGGCCACACAGTCACGCGCTCTGGCGATTCGCTCCAACGAACTGGCTGACAGTGCATCCACTAAAGTTACCGAGGCTGCCCGTGTGGCAAACCAAGCTCAACAGCTTTCCAAATTCTTTGAGTAATCAAACA‑GGAGAAACCATTATGATCGTTTCTGACATCGAAGCTAACGCCCTCTTAGAGAG                                                                                                                                                                                                                                                                        <  1:296992/300‑1`  
                                               `acagCACTAAAGGAGACACACCATGTTCAAACTGATTAAGAAGTTAGGCCAACTGCTGGTTCGTATGTACAACGTGGAAGCCAAGCGACTGAACGATGAGGCTCGTAAAGAGGCCACACAGTCACGCGCTCTGGCGATTCGCTCCAACGAACTGGCTGACAGTGCATCCACTAAAGTTACCGAGGCTGCCCGTGTGGCAAACCAAGCTCAACAGCTTTCCAAATTCTTTGAGTAATCAAACA‑GGAGAAACCATTATGATCGTTTCTGACATCGAAGCCAACGCCCTCTTAGAGAGCGTCAC                                                                                                                                                                                                                                                                  <  1:289038/297‑1`  
                                               `aACTCACTAAAGGAGACACACCATGTTCAAACTGATTAAGAAGGTAGGCCAACTGCTGGTTCGTATGTACAACGTGGAAGCCAAGCGACTGAACGATGAGGATCGTAAAGAGGCCACACAGTCACGCGCTCTGGCGAGTCGCTCCAACTAACTGGCTGACAGTGCATCCACTAAAGTTACCGAGGCTGCCCGTGTGGCAAACCAAGCTCAACAGCTTTCCAAATTCTTTGAGTAATCAAACA‑GGAGAAATCAATATGATCGTTTCTGACATCGAAGCTAACGCCCTCTTAGAGAGCGTCAC                                                                                                                                                                                                                                                                  <  1:453379/300‑1`  
                                                `ACTCACTACAGGAGACACACCATGTTCAAACTGATTAAGAAGTTAGGCCAACTGCTGGTTCGTATGTACAACGTGGAAGCCAAGCGACTGAACGATGAGGCTCGTAAAGAGGCCACACAGTCACGCGCTCTGGCGATTCGCTCCAACGAACTGGCTGACAGTGCATCCACTAAAGTTACAGAGGCTGCCCGTGTGGCAAACCAAGCTCAACAGCTTTCCACATTCTTTGAGTAATCAAACA‑GGAGAAACCATTATGATCGTTTCTGACATCGAAGCTAACGCCCTCTTAGAGAGCGTCACT                                                                                                                                                                                                                                                                 >  1:363541/1‑301`  
                                                `ACTCACTAAAGGAGACACACCATGTTCAAACTGATTAAGAAGTTAGGCCAACTGCTGGTTCGTATGTACAACGTGGAAGCCAAGCGACTGAACGATGAGGCTCGTAAAGAGGCCACACAGTCACGCGCTCTGGCGATTCGCTCCAACGAACTGGCTGACAGTGCATCCACTAACGTTACCGAGGCTGCCCGTGTGGCAAACCAAGCTCAACAGCTTTCCAAATTCTTTGAGTAATCAAACA‑GGAGAAACCACTATGAACGTTTCTGAACTCGAAGCTAACGCTCTCTTAGAGAGCGTCcat                                                                                                                                                                                                                                                                 >  1:269097/1‑298`  
                                                   `CACTAAAGGAGACACACCATGTTCAAACTGATTAAGAAGTTAGGCCAACTGCTGGTTCGTATGTACAACGTGGAAGCCAAGCGACTGAACGATGAGGCTCGTAAAGAGGCCACACAGTCACGCGCTCTGGCGATTCGCTCCAACGAACTGGCTGACAGTGCATCCACTAAAGTTACCGAGGCTGCCCGTGTGGCCAACCAAGCTCAACAGCTTTCCAAATTCTTTGAGTAATCAAACA‑GGAGAAATCAATATGATCGTTTCTGACATCGAAGCTAACGCCCTCTTAGAGAGCGTCACTAAG                                                                                                                                                                                                                                                              >  1:441057/1‑301`  
                                                   `CACTAAAGGAGACACACCATGTTCAAACTGATTAAGAAGTTAGGCCAACTGCTGGTTCGTATGTACAACGTGGAAGCCAAGCGACTGAACGATGAGGCTCGTAAAGAGGCCACACAGTCACGCGCTCTGGCGATTCGCTCCAACGAACTGGCTGACAGTGCATCCACTAAAGTTACCGAGGCTGCCCGTGTGGCAAACCAAGCTCAACAGCTTTCCAAATTCTTTGAGTAATCAAACA‑GGAGAAACCATTATGATCGTTTCTGACATCGAAGCTAACGCCCTCTTAGAGctgtctcttata                                                                                                                                                                                                                                                              >  1:167779/1‑289`  
                                                    `ACTAAAGGAGACACACCATGTTCAAACTGATTAAGAAGTTAGGCCAACTGCTGGTTCGTATGTACAACGTGGAAGCCAAGCGACTGAACGATGAGGCTCGTAAAGAGGCCACACAGTCACGCGCTCTGGCGATTCGCTCCAACGAACTGGCTGACAGTGCATCCACTAAAGTTACCGAGGCTGCCCGTGTGGCAAACCAAGCTCAACAGCTTTCCAAATTCTTTGAGTAATCAAACA‑GGAGAAACCATTATGATCGTTTCTGACATCGAAGCTAAAGCCCTCTTAGAGAGCGTCATTAAGT                                                                                                                                                                                                                                                             >  1:47387/1‑301`  
                                                    `ACTAAAGGAGACACACCATGTTCAAACTGATTAAGAAGTTAGGCCAACTGCTGGTTCGTATGTACAACGTGGAAGCCAAGCGACTGAACGATGAGGCTCGTAAAGAGGCCACACAGTCACGCGCGCTGGCGATTCGATCCAACGAACTGGCTGACAGTTCAGCCACTACAGTTACCGAGGCTGCCCGTGTGGCAAACCAAGCTAAACAGCTTTCCAAATTCTTTGAGTAATCAAACC‑GGAGAAACCATTAGGATCGTTTATGACATCGAAGCTAACGCCCTCTTAGAGAGatggctcttat                                                                                                                                                                                                                                                             >  1:332837/1‑290`  
                                                          `tcggagatgtgtataagagacaGATTAAGAAGTTAGGCCAACTGCTGGTTCGTATGTACAACGTGGAAGCCAAGCGACTGAACGATGAGGCTCGTAAAGAGGCCACACAGTCACGCGCTCTGGCGATTCGCTCCAACGAACTGGCTGACAGTGCATCCACTAAAGTTACCGAGTCTGCACGTGT‑GCAAACCAAGCTCAACAGCTTTCCAAATTCTTTGAGTAATCAAACA‑GGAGAAATCAATATGATCGTTTCTGACATCGAAGCTAACGACCTCTTAGAGAGCGTCACTAAGTTCCACTG                                                                                                                                                                                                                                                      <  1:11327/279‑1`  
                                                          `GGAGACACACCATGTTCAAACTGATTAAGAAGTTAGGCCAACTGCTGGTTCGTATGTACAACGTGGAAGCCAAGCGACTGAACGAGGAGGCTCGTAAAGAGGCCACACAGTCACGCGCTCTGGCGATTCGCTCCAACGAACTGGCTGACAGTGCATCCACTAAAGTTACCGAGGCTGCCCGTGTGGCAAACCAAGCTCAACAGCTTTCCGAATTCTTTGAGTAATCAAACA‑GGAGAAACCATTCTGATCGTTTCTTACATCAAAGCTAACGCCCTCTTGGAGAGCGTCACTAATTTCCACT                                                                                                                                                                                                                                                       >  1:208223/1‑301`  
                                                                 `accaATGTTGACACTGAGTACGAAGTTAGGCCAACTGCTGGTTCGCATGTACAACGTGGAAGCACAGCGAATGAACGATGAGGCTCGGAAAGAGGCCACACAGTCACGCGCTCTGGCGATTCGCTCCAACGAACTGGCTGACAGTGCATCCACTAAAGTTACCGAGGCTGCCAGTGTGGCAAACCAAGCTCAACAGCTTTCCAAATTCTTTGAGTAATCAAACA‑GGAGAAACCATTATGATCGTTTCTGACATCGAAGCTAACGCCCTCTTAGAGAGCGTCACTAAGTTCCACTGCGGGGT                                                                                                                                                                                                                                                <  1:492526/297‑1`  
                                                                      `TGTTCAAACTGATTAAGAAGTTAGGCCAACTGCTGGTTCGTATGTACAACGTGGAAGCCAAGCGACTGAACGATGAGGCTCGTAAAGAGGCCACATAGTCACGCGCTCTGGCGATTCGCTCCAACGAACTGGCTGACAGTGCATCCACTAAAGTTACCGAGGCTGCCCGTGTGGCAAACCAAGCTCAACAGCTTTCCCAATTCTTTGAGTAATCAAACA‑GGAGAAATCAATATGATCGTTTCTGACATCGAAGCTAACGCCCTCTTAGAGAGCGTCACTAAGTTCCACTGCGGGGTTATCT                                                                                                                                                                                                                                           >  1:436756/1‑301`  
                                                                       `GTTCAAACTGATTAAGAAGTTAGGCCAACTGCTGGTTCGTATGTACAACGTGGAAGCCAAGCGACTGAACGATGAGGCTCGTAAAGAGGCCACACAGTCACGCGCTCTGGCGATTCGCTCCAACGAACTGGCTGACAGTGCATCCACTAAAGTTACCGAGGCTGCCCGTGTGGCAAACCAAGCTCAACAGCTTTCCAAATTCTTTGAGTAATCAAACC‑GGAGAAACCATTATGATCGTTTCTGACATCGAAGCTAACGCCCTCTTAGAGAGCGTCACTAAGTTCCACTGCGGGGTctgtct                                                                                                                                                                                                                                          >  1:109691/1‑295`  
                                                                        `taCAAACTGATTCAAGAGTTAGGCCAACTGCTGGTTCGTATGTACAACGTGGAAGCCAAGCGACTGAACGATGAGGTTCGTAAAGATGCCACACGGTCACGCGCTCTGGCGGTTCGCTCCAACGAACTGGCTGACAGTGCATCCACTAAAGTTACCGAGGCGGCCCGTGTGGCAAACCAAGATCAACAGCTTTCCAAATTCTTTGAGTAATCAAACA‑GGAGAAACCATTATGATCGTTTCTGACATCGAAGCTAACGCCCTCTTAGAGAGCGTCACTAAGTACCACTGCGGGGTTATCTAC                                                                                                                                                                                                                                         <  1:469932/299‑1`  
                                                                        `TTCAAACTGATTAGGAAGTTAGGCCAACTGCTGGTTCGTATGTACAACGTGGAAGCCAAGCGACTGAACGATGAGTCTCGTAAAGAGGCCACACAGTCACGCGCTCTGGCGATTCGCTCCAACGAACTGGCTGACAGTGCATCCACTAAAGTTACCGAGGCTGCCCGTGTGGCAAACCAAGCTCAACAGCTTTCCAAATTCTTTGAGTAATCAAACA‑GGAGAAACCATTATGATAGTTTCTGACATCGAAGCTAACGCCCTCTTAGAGAGCGTCACTAAGTTCCACTGCGGGGTTATCTAC                                                                                                                                                                                                                                         <  1:272445/301‑1`  
                                                                           `ataagagacagGAAGTTAGGCCAACTGCTGGTTCGTATGTACAACGTGGAAGCCAAGCGACTGAACGATGAGGCTCGTAAAGAGGCCACACAGTCACGCGCTCTGGCGATTCGCTCAAACGAACTGGCTGACAGTGCATCCACTAAAGTTACCGAGGCTGCCCGTGTGGCAAACCAAGCTCAACAGCTTTCCAAATTCTTTGAGTAATCAAACA‑GGAGAAACCATTATGATCGTTTCTGACATCGAAGCTAACGCCCTCTTAGAGAGCGTCACTAAGTTCCACTGCGGGGTTATCTACGAC                                                                                                                                                                                                                                      <  1:221248/290‑1`  
                                                                           `ataagagaaagGAAGTTAGGCCAACTGCTGGTTCGTATGTACAACATGGAAGCCAAGCGACTGAACGATGAGGCTCGTAAAGAGGCCACACAGTCACGCGCTCTGGCGATTCGCTCCAACGAACTGGCTGACAGTGCATCCACTAAAATTACCGAGGCTGCCCGTGTGGCAAACCAAGCTCAACAGCTTTCCAAATTCTTTGAGTAATCAAACA‑GGAGAAACCATTATGATCGTTTCTGACATCGAAGCTAACGCCCTCTTAGAGAGCGTCACTAAGTTCCACTGCGGGGTTATCTACGAC                                                                                                                                                                                                                                      <  1:306238/290‑1`  
                                                                           `agacaGATTAAGAAGTTAGGCCCACAGCTGGTTCGTATGTACAACGTGGAAGCCAAGCGACTGAACGATGAGGCTCGTAAAGAGGCCACACAGTCACGCGCTCTGGCTATTCGCTCCAACGAACTGGCTGACAGTGCATCCACTAAAGTTACCGAGGCTGCCCGTGTGGCAAACCAAGCTCAACAGCTTTCCAAATTCTTTGAGTAATCAAACA‑GGAGAAACCATTATGATCGTTTCTGACATCGAAGCTAACGCCCTCTTAGAGAGCGTCACTAAGTTCCACTGCGGGGTTATCTACGAC                                                                                                                                                                                                                                      <  1:224834/296‑1`  
                                                                           `AAACTGATTAATGAGTTAGGCAAACTGATGGTTCGGATGTACAACGTGGAAGCCAAGCGACGGAACGATGAGGCTCGTAAAGAGGCCACACAGTCACGCGCTGTGGCGATTAGATCCAACGAACTGGCTGACAGTGCATCCACTAAAGTTACCGAGGCTGCCCGTGTGGCAAACCAAGCTCAACAGCTTTCCAAATTCTTTGAGTAATCAAACA‑GGAGAAACCAATATGATCGTTTCTGACATCGAAGCTAACGCCCTCTTAGAGAGCGTCACTAAGTTCCACTGCGGGGTTATCTACGAC                                                                                                                                                                                                                                      <  1:5271/301‑1`  
                                                                           `AAACTGATTAAGAAGTTAGGCCAACTGCTGGTTCGTATGTACAACGTGGAAGCCAAGCGACTGAACGATTAGGCTCGTAAAGAGGCCACATAGTCACGCGCTCTGGCGATTCGCTCCAACGAACTGGCTGACAGTGCATCCACTAAAGTTACCGAGGCTGCCCGTGTGGCAAACCAAGCTCAACAGCTTTCCAAATTCTTTGAGTAATCAAACA‑GGAGAAACCATTATGATCGTTTCTGACATCGAAGCTAACGCCCTCTTAGAGAGCGTCACTAAGTTCCACTGCGGGGTTATCTACGAC                                                                                                                                                                                                                                      <  1:72539/301‑1`  
                                                                           `AAACTGATTAAGAAGTTAGGCCAACTGCTGGTTCGTATGTACAACGTGGAAGCCAAGCGACTGAACGATGAGGCTCGTAAAGAGGCCACACAGTCACGCGCTCTGGCGATTCGCTCCAACGAACTGGCTGACAGTGCATCCACTAAAGTTACCGAGGCTGCCCGTGTGGCAAACCAAGCTCAACAGCTTTCCAAATTCTTTGAGTAATCAAACA‑GGAGAAACCATTATGATCGTTTCTGACATCGAAGCTAACGCCCTCTTAGAGAGCGTCACTAAGTTCCACTGCGGGGTTATCTACGAC                                                                                                                                                                                                                                      <  1:147220/301‑1`  
                                                                           `AAACTGATTAAGAAGTTAGGCCAACTGCTGGGTCGTATGGACAACGTGGAAGCCAAGCGACTGAACGATGAGGCTCGTAAAGAGGCCACACAGTCACGCTCTCTGGCGATTCGCTCCAACGAACTGGCTGACAGTGCATCCACTAAAGTTACCGAGGCTTCCCGTGTGGCAAACCAAGCTCAACAGCTTTCCAAATTCTTTGAGTAATCAAACA‑GGAGAAACCATTATGATCGTTTCTGACATCGAAGCTAACGCCCTCTTAGAGAGCGTCACTAAGTTCCACTGCGGGGTTATCTACGAC                                                                                                                                                                                                                                      <  1:326920/301‑1`  
                                                                              `CTGATTAAGAAGTTAGGCCAACTGCTGGTTCGTATGTACAACGTGGAAGCCAAGCGACTGAACGATGAGGCTCGTAAAGAGGCCACACAGTCACGCGCTCTGGCGATTCGCTCCAACGAACTGGCTGACAGTGCATCCACTAATGTTACCGAAGCTGCCCGTGTGGCAAACCACGCTCAACAGCTTTCCAAATTCTTTGAGTAATCAAACA‑GGAGAAACAATTATGATCGTATCTTACATCGAAGCTAACGCCCTCTTAGATAGCGCCCCTAAGTTCCCCTGCGGGGTTATCTACGACTAC                                                                                                                                                                                                                                   >  1:80947/1‑301`  
                                                                                      `tgataagagaccgCTGCTGGTTCGTATGTACACCGTCGCAGCCAAGCGACTGAACGATGAGGCTCGTAAAGAGGCCACACAGTCACGCGCTCTGGCGATTCTCTCCAACGAACTGGCTGACAGTGCATCCACTAAAGCTACCGAGGCTGCCCGTGTGGCAAACCAAGCTCAACAGCTTTCCAAATTCTTTGAGTAATCAAACA‑GGAGAAATCAATATGATCGTTTCTGACATCGAAGCTAACGCCCTCTTAGAGAGCGTCACTAAGTTCCACTGCGGGGTTATCTACGACTACTCCACCGC                                                                                                                                                                                                                           <  1:368641/288‑1`  
                                                                                        `AGTTAGGCCAACTGCTGGTTCGTATGTACAACGTGGAAGCCAAGCGACTGAACGATGAGGCTCGTAAAGAGGCCACACAGTCACGCGCTCTGGCGATTCGCTCCAACGAACTGGCTGACAGTGCATCCACTAAAGTTACCGAGGCTGCCCGTGTGGCAAACCAAGCTCAACAGCTTTCCAAATTCTTTGAGTAATCAAACA‑GGAGAAACCATTATGATCGTTTCTGACATCGAAGCTAACTCCCTCTTAGAGAGCGTCACTAAGTTCCACTGCGGGGTGATCTACGACTACTCCACCGCct                                                                                                                                                                                                                         >  1:292966/1‑299`  
                                                                                                      `CTGGATCGAATGTACAACGGGGAAGCCAAGCGACTGAACGATGAGGCTCGTAAAGAGGCCACACAGTCACGCGCTCGGGCGATTCGATCCAACGAACTGGCTGACAGTGCATCCACTAAAGTTACCTAGGCTGCCCGTGTGGCAAACCAGGCGCAACTGCTTTCCAAATTCTTGGAGTAATCAAACA‑GGAGAAACCATTATGATCGTGTCTGACATCGAAGCTAACGCCCTCTTAGAGAGCGTCACTAAGTTCCACTGCGGGGTTATCTACGACTACTCCACCGCTGAGTACGTAAGCTAC                                                                                                                                                                                                           <  1:27761/301‑1`  
                                                                                                          `agCGTATGTACAACGTGGAAGCCAAGCGACTGAACGATGAGGCTCGTAAAGAGGCCACACAGTCACGCGCTCTGGCGATTCGCTCCAACGAACTGGCTGACAGTGCATCAACTAAAGTTACCGAGGCTGCCCGTGTGGCAAACCAAGCTCAACAGCTTTCCAAATTCTTTGAGTAATCAAACA‑GGAGAAACCATTATGATCGTTTCTGACATCGAAGCTAACGCCCTCTTAGAGAGCGTCACTAAGTTCCACTGCGGGGTTATCTACGACTACTCCACCGCTGAGTACGTAAGCTACCGTC                                                                                                                                                                                                       <  1:387343/299‑1`  
                                                                                                          `TTCGTATGTACAAAGTTGAAGCCCAGCGCCTGAACGTTGAGGCTCGTAAAGAGGCCACACAGTCCCGCGCTCTGGCGATTCGCTCCAACGAACTGGCTGACAGTGCATCCACTAAAGTTACCGAGGCTGCCCGTGTGGCAAACCAAGCTCAACAGCTTTCCAAATTCTTTGAGTAATCAAACA‑GGAGAATCCATTATGATCGTTTCTGACATCGAAGCTAACGCCCTCTTAGAGAGCGTCACTAAGTTCCACTGCGGGGTTATCTACGACTACTCCACCGCTGAGTACGTAAGCTACCGTC                                                                                                                                                                                                       <  1:508889/301‑1`  
                                                                                                              `TATTTACAACCTGGAAGCCAAGCGACTGAACGATGAGGCTCGTAAAGAGGCCACACAGTCACGCGCTCTGGCGAGTCGCTCCAACGAACTGGCTGACAGTGCATCCACTAAAGTTACCGAGGCTGCCCGTGTGGCAAACCAAGCTCAACAGCTTTCCAAATTCTTTGAGTAATCAAACA‑GGAGAAATCAATATGATCGTTTCTGACATCGAAGCTAACGCCCTCTTAGAGAGCGTCACTAAGTTCCACTGCGGGGTTATCTACGACTACTCAACAGCTGAGTACGTAAGCTACCGTCCGAG                                                                                                                                                                                                   <  1:19445/301‑1`  
                                                                                                                `TGTACAACGTGGAAGCCAAGCGACTGAACGATGAGGCTCGTAAAGAGGCCACACAGTCACGCGCTCTGGCGATTCGCTCCAACGAACTGGCTGACAGTGCATCCACTAAAGTTACCGAGGCTGCCCGTGTGGCAAACCAAGCTCAACAGCTTTCCAAATTCTTTGAGTAATCAAACA‑GGAGAAACCATTATGATCGTTTCTGACATCGAAGCTAACGCCCTCTTAGAGAGCGTCACTAAGTTCCACTGCGGGGTTATCTACGACTACTCCACCGCTGAGTACGTAAGCTACCtgtctctta                                                                                                                                                                                                 >  1:263304/1‑292`  
                                                                                                                  `TACAACGTGGAAGCCAAGCGACTGAACGATGGGGCTCGTAAAGAGGCCACACAGTCACGCGCTCTGGCGATTCGCTCCAACGAACTGGCTGACAGTGCATCCACTAAAGTTACCGAGGCTGCCCGTGTGGCAAACCAAGCTCAACAGCTTTCCAAATTCTTTGAGTAATCAAACA‑GGAGAAACCATTATGATCGTTTCTGACATCGAAGCTAACGCCCTCTTAGAGAGCGTCACTAAGTTCCACTGCGGGGTTATCTACGACTACTCCACCGCTGAGTACGTAAGCTACCGTCCGAGTGAC                                                                                                                                                                                               <  1:133930/301‑1`  
                                                                                                                    `aAACGTGGAAGCCAAGCGACTGAAAGATGAGGCTCGTAAAGAGGCCACACAGTAACGCGCTCTGGCGATTCGCTCAAACGAACTGGCTGACAGTGCATCCACTAAAGTTACCGAGGCTGCCCGTGTGGAAAACCAAGCTCAACAGCTTTCCAAATTCTATGAGTAATCAAACA‑GGAGAAACCATTATGATCGTTTCTGACATCGAAGCTAACGCCCTCTGAGAGAGAGTCACTAAGTTCCACTGCGGGGTTATCTACGACTACTCCACCGCTGAGTACGTAAGCTACCGTCCGAGTGACTT                                                                                                                                                                                             <  1:419457/300‑1`  
                                                                                                                       `CGTGGAAGCCAGGCGACTGAACGATGAGGCTCGTAAAGAGGCCACACAGTCACGCGCTCTGGCGATTCGCTCCAACGAACTGGCTGACAGTGCATCCACTAAAGTTACCGAGGCTGCCCGTGTGGCAAACCAAGCTCGACAGCTTTCCAAATTCTTTTAGTAATCAAACA‑GGAGAAACCATTATGATCGTTTCTGACATCGAAGCTAACGCCCTCTTAGAGAGCGTCACTAAGTTCCACTGCGGGGTTATCTACGAATACTACACCGCTGAGTACGTAAGCTACCGTCCGAGTGACTTCGG                                                                                                                                                                                          <  1:310156/301‑1`  
                                                                                                                       `CGTGGAAGCCAAGCGACTGAACGATGAGGCTCGTAAAGAGGCCACACAGTCACGCGCTCTGGCGATTCGCTCCAACGAACTGGCTGACAGTGCATCCACTAAAGTTACCGAGGCTGCCCGTGTGGCAAACCAAGCTCAACAGCTTTCCAAATTCTTTGAGTAATCAAACA‑GGAGAAACCATTATGATCGTTTCTGACATCGAAGCTAACGCCCTCTTAGAGAGCGTCACTAAGTTCCACTGCGTGGTTATCTACGACTACTCCACCGCTGCGTACGTAAGCTACCGTCCGAGTGACTTCGG                                                                                                                                                                                          >  1:39268/1‑301`  
                                                                                                                         `TGGAAGCCAAGCGACTGAACGATGAGGCTCGTAAAGAGGCCACACAGTCACGCGCTCTGGCGATTCGCTCCAACGAACTGGCTGACAGTGCATCCACTAAAGTTACCGAGGCTGCCCGTGTGGCAAACCAAGCTCAACAGCTTTCCAAATTCTTTGAGTAATCAAACA‑GGAGAAACCATTATGATCGTTTCTGACATCGAAGCTAACGCCCTCTTAGAGAGCGTCACTAAGTTCCACTGCGGGGTTATCTACGACTACTCCACCGCTGAGTACGTAAGCTACCGTCCGAGTGACTTCGGTG                                                                                                                                                                                        <  1:396339/301‑1`  
                                                                                                                              `GCCAAGCGACTGGACGATGAGGCTCGTAATGAGGCCACACAGTCACGCGCTCTGGCGATTCGCTCCAACGAACTGGCTGACAGTGCATCCACTAAAGTTTCCGATGCTGCACGTGTGGCAAACCAAGCTCAACGGCTTTTCAAAGTCTTTGAGTAATCAAACA‑GGAGCAAACTTTGTGATCGTTTCTGACATCGAAGCTAACGCCCTCTTATAGAGCGTCATTACGTTACACTGCGGGGTTATCTACGACTACTCCACTGCTTAGTCCGTGAGCTACCGTCCGAGTGCCTTCGGTGCGTtt                                                                                                                                                                                   >  1:432538/1‑299`  
                                                                                                                                 `AAGCGACTGAACGATGAGGATCGTAAAGAGGCCACACAGTCACGCGCTCTGGCGATTCGCTCCAACGAACTGGCTGACAGTGCATCCACTAAAGTTACCGAGGCTGCCCGTGTGGCAAACCAAGCTCAACAGCTTTCCAAATTCTTTGAGTAATCAAACA‑GGAGAAACCATTATGATCGTTTCTGACATCGAAGCTAACGCCCTCTTAGAGAGCGTCACTAAGTTCCACTGCGGGGTTATCTACGACTACTCCACCGCTGAGTACGTAAGCTACCGTCCGAGTGACTTCGGTGCGTATCTG                                                                                                                                                                                <  1:382064/301‑1`  
                                                                                                                                   `GCGACTGAACGATGAGGCCCGTAAAGAGGCCACACAGTCACGCGCTCTGGCGATTCGCTCCAACGAACTGGCTGACAGTGCATCCACTAAAGTTACCGAGGCTGCCCGTGTGGCAAACCAAGCTCAACAGCTTTCCAAATTCTTTGAGTAATCAAACA‑GGAGAAACCATTATGATCGTTTCTGACATCGAAGCTAACGCCCTCTTAGAGAGCGTCACTAAGTTCCACTGCGGGGTTATCTACGACTACTACACCGCTGAGTACGTAAGCTACCGTCCGAGTGACTTCGGTGCGTATCTGGA                                                                                                                                                                              <  1:121570/301‑1`  
                                                                                                                                    `CGACTGAACGATGAGGCTCGTAAAGAGGCCACACAGTCACGCGCTCTGGCGATTCGCTCCAACGAACTGGCTGACAGTGCATCCACTAAAGTTACCGAGGCTGCCCGTGTGGCAAACCAAGCTCAACAGCTTTCCAAATTCTTTGAGTAATCAAACA‑GGAGAAACCATTATGATCGTTTCTGACATCGAAGCTAACGCCCTCTTAGAGAGCGTCACTAAGTTCCACTGCGGGGTTATCTACGACTACTCCACCGCTGAGTACGTAAGCTACCGTCCGAGTGACTTCGGTGCGTATCTGGAT                                                                                                                                                                             <  1:479759/301‑1`  
                                                                                                                                        `agagCGAGGAGGCTCGTAAAGAAGCCACACAGGTACGCGCTCTGGCGAATCGCTCCAACGAACTGGCTGACAGAGCATCCACTAAAGTCACCGATGCTGCCCGTGTGGCAAACCAAGCTCAACAGCTTTCCAAATTCGTGGAGTAATCAAACA‑GGAGAATCCATTATGATCGTTTCTGACATCGAAGCTAACGCCCTCTTAGAGAGCGTCACTAAGTTCCACTGCGGGGTTATCTACTACTACTCCACCGCTGAGTACGTAAGCTACCGTCCGAGTGACTTCGGTGCGTATCTGGATGCGC                                                                                                                                                                         <  1:498662/297‑1`  
                                                                                                                                         `GAACGATGAGGCTCGTAAAGAGGCCACACAGTCACGCGCTCTGGCGATTCGCTCCAACGAACTGGCTGACAGTGCATCCACTAAAGTTACCGAGGCTGCCCGTGTGGCAAACCAAGCTCAACAGCTTTCCAAATTCTTTGAGTAATCAAACA‑GGAGAAATCAATATGATCGTTTCTGACATCGAAGCTAACGCCCTCTTAGAGAGCGTCACTAAGTTCCACTGCGGGGTTATCTACGACTACTCCACCGCTGAGTACGTAAGCTACCGTCCGAGTGACTTCGGTGCGTATCTGGATGCGCT                                                                                                                                                                        >  1:198957/1‑301`  
                                                                                                                                                   `aagagacagGAGGCCACACAGTCACGAGCTCTGGCGATTCGCTCCAACGAACTGGCTGACAGTGCATCCACTAAAGTTACCGAGGCTGCCCGTGTGGCAAACCAAGCTCAACAGCTTTCCAAATTCTTTGAGTAATCAAACA‑GGAGAAACCATTATGATCGTTTCTGACATCGAAGCTAACGACCTCTTAGAGAGCGTCACTAAGTTCCACTGCGGGGTTATCTACGACTACTCCACCGCTGAGTACGTAAGCTACCGTCCGAGTGACTTCGGTGCGTATCTGGATGCGCTGGAAGCCGAG                                                                                                                                                              <  1:248165/292‑1`  
                                                                                                                                                          `cAGAGGCCACACGGTCACGCGCTCTGGCTATTCGCTCCAACGAACTGGCTTACAGTGCATCCACTAAAGTTCCCGAGCCTGCCCGTGTTGCAAACCAAGCTCAACAGCTTTCCAAATTCGTTGAGTACTCAAACA‑GGAGAAACCATTATGATCGATTCTGACAGCGAAGCTAACGCCCTCTTAGAGAGCGTCACTAAGTTCCACTGCGGGGTTATCTACGACTACTCCACCGCTGAGTACGTAAGCTACCGTCCGAGTGACTTCGGTGCGTATCTGGATGCGCTGGAAGCCGAGGTTGCAC                                                                                                                                                       <  1:243963/300‑1`  
                                                                                                                                                          `AAGGGGACACACAGTCACGCGCTCTGGCGATTCGCGTCAACGAACTGGCGGACAGTGCATCCACTAAAGTTACCGAGGCTGCCCGTGTGGCAAACCAAGCTCAACAGCTTTCCAAATTCTTTGAGTAATCAAACA‑GGAGAAACCATTATGATCGTTTCTGACATCGAAGCTAACGCCCTCTTAGAGAGCGTCACTAAGTTCCACTGCGGGGTTATCTACGACTACTCCACCGCTGAGTACGTAAGCGACCGTCCGAGTGACTTCGGTGCGTATCTGGATGCGCTGGAAGCCGAGGTTGCAC                                                                                                                                                       <  1:46215/301‑1`  
                                                                                                                                                            `GAGGCCACACAGTCACGCGCTATGGCGATTCGCTCCAACGAACTGGCTGACAGTGCATCCACTAAAGTTACCGAGGCTGCCCGTGTGGCAAACCAAGCTCAACAGCTTTCCAAATTCTTTGAGTAATCAAACA‑GGAGAAACAATTATGATCGTTTCTGACATCGAAGCTAACGCCATCTTAGAGAGCGTCACTAAGTTCCACTGCGGGGTTATCTACTACTACTACACCGCTGAGTACGTAAGCTACCATCCTAGTGACTTCGTTGCGTATCTGGATGCGATGGAAGCCGAGGTTGCccct                                                                                                                                                     >  1:281457/1‑297`  
                                                                                                                                                                   `agCAGTCACGCGCGCTGGCGATTCGCTCCAACGAACTGGCTGACAGTGCATCCACTAAAGTTACCGAGGCTGCCCGTGTGGCAAACCAAGCTCAACAGCTTTCCAAATTCTTTGAGTAATCAAACA‑GGAGAAATCAATATGATCGTTTCTGACATCGAAGCTAACGCCCTCTTAGAGAGCGTCACTAAGTTCCACTGCGGGGTTATCTACGACTACTCCACCGCTGAGTACGTAAGCTACCGTCCGAGTGACTTCGGTGCGTATCTGGATGCGCTGGAAGCCGAGGTTGCACGAGGCGGTC                                                                                                                                              <  1:91271/299‑1`  
                                                                                                                                                                         `agacatgtatgaCGATTCGCGCTAACGAACTGGCTGCAAGTGCAGTCACTAAAGTTACCGAGGCTGCCCGTGTGGCAAACCAAGCTCAACAGCTTTCCAAATTCTTCGAATAATCAAACA‑GGAGAATCCATTATGATCGTTTCTTACATCGAAGCTAACGCCCTCTTCGAGAGCGTCAATAAGTTCCACTGCGGGGTTATCTACGACTACTCCACCGCTGATTACGTAAGCTACCGTCCGAGTGACTTCGGTGCGTATCTGGATGCGCTGGAAGCCGAGGTTGCACGAGGCGGTCTTATTG                                                                                                                                        <  1:503804/289‑1`  
                                                                                                                                                                         `CACGCGCTCTGGCGATTCGCTCCAACGAACTGGCTGACAGTGCATCGACTAAAGTTACCGAGTCTGCCCGTGTGGCAAACCAAGCTCAACAGCTTTCCAAATTCTTTGAGTAATCAAACA‑GGAGAAACCATTATGATCGTTTCTGACATCGAAGCTAACGCCCTCTTAGAGAGCGTCACTAAGTTCCACTGCGGGGTTATCTACGACTACTCCACCGCTGAGTACGTAAGCTACCGTCCGAGTGACTTCGGTGCGTATCTGGATGCGCTGGAAGCAGAGGTTGCACGAGGCGGTCTTATTG                                                                                                                                        <  1:143212/301‑1`  
                                                                                                                                                                         `CACGCGCTCTGGCGATTCGCTCCAACGAACTGGCTGACAGTGCATCCACTAAAGTTACCGAGGCTGCCCGTGTGGCAAACCAAGCTCAACAGCTTTCCAAATTCTTTGAGTAATCAAACA‑GGAGAAATCAATATGATCGTTTATGACATCGAAGCTAACGCCCTCTTAGAGAGCGTCACTAAGTTCCACTGCGGGGTTATCTACGACTACTCCACCGCTGAGTACGTAAGCTACCGTCCGAGTGACTTCGGTGCGTATCTGGATGCGCTGGAAGCCGAGGTTGCACGAGGCGGTCTTATTG                                                                                                                                        <  1:1569/301‑1`  
                                                                                                                                                                         `CACGCGCTCTGGCGATTCGCTCCAACGAACTGGCTGACAGTGCATCCACTAAAGTTACCGAGGCTGCCCGTGTGGCAAACCAAGCTCAACAGCTTTCCAAATTCTTTGAGTAATCAAACA‑GGAGAAACCATTATGATCGTTTCTGACATCGAAGCTAACGCCCTCTTAGAGAGCGTCACTAAGTTCCACTGCGGGGTTATCTACGACTACTACACCGCTGAGTACGTAAGCTACCGTCCGAGTGACTTCGGTGCGTATCTGGATGCGCTGGAAGCCGAGGTTGCACGAGGCGGTCTTATTG                                                                                                                                        <  1:215467/301‑1`  
                                                                                                                                                                         `CACGCGATCTGCGGATTCGCTCCAACGAACTGGCTGACAGTTCATCCACTAAAGTTACCGAGGCGGCCCGTGTGGCAAACCAAGCTCAACAGCTTTCCAAATTCTTTGAGTAATCAAACA‑GGAGAAACCATTATGATCGTTTCTGACATCGAAGCTAACGCCCTCTTAGAGAGCGTCACTAAGTTCCACTGCGGGGTTATCTACGACTACTCCACCGCTGAGTACGTAAGCTACCGTCCGAGTGACTTCGGTGCGTATCTGGATGCGCTGGAAGCCGAGGTTGCACGAGGCGGTCTTATTG                                                                                                                                        <  1:184859/301‑1`  
                                                                                                                                                                                 `CTGGCGATTCGCTCCAACGAACTGGCTGACAGTGCATCCACTAAAGTTACCGAGGCTGCCCGTGTGGCAAACCAAGCTCAACAGCTTTCCAAATTCTTTGAGTAATCAAACA‑GGAGAAACCATTATGAACGTTTCTGACATCGAAGCTAACGCCCTCTTAGAGAGCGTCACTAAGTTCCACTGCGGGGTTATCTACGACTACTCCACCGCTGAGTACGTAAGCTACCGTCCGAGTGACTTCGGTGCGTATCTGGATGCGCTGGAAGCCGAGGTTGCACGAGGCGGTCTTATTGctgtctct                                                                                                                                >  1:314764/1‑293`  
                                                                                                                                                                                   `GGCGATTCGCTCCAACGAACTGGCTGACAGTGCATCCACTAAAGTTACCGAGGCTGCCCGTGTGGCAAACCAAGCTCAACAGCTTTCCAAATTCTTTGAGTAATCAAACA‑GGAGAAATCAATATGATCGTTTCTGGCCTCGAAGCTAACGCCCTCTTAGAGATCGTCACTAAGTTCCACTGCGGGGTTATCTACGACTACTCCACCGCTGAGTACGTAAGCTACCGTCCGAGTGACATCGGTGCGTATCCGGATGCGCTGGAAGCCGAGCTTGAACGAGGCctgcctcttatacccatctc                                                                                                                              >  1:238056/1‑281`  
                                                                                                                                                                                   `GGCGATTCGATCCAACGAACTGGATGACAGTGCATCCACTAAAGTTACCGAGGCTGCCCGTGTGGAAAACCAAGCTCAACAGCTTTCCAAATTCTTTGAGTAATCAAACA‑GGAGAAACCATTATGATCGTTTCTGACATCGAAGCTAACGCCCTCTTAGAGAGCGTCACTAAGTTCCACTGCGGGGTTATCTACGACTACTCCACCGCTGAGTACGTAAGCTACCGTCCGAGTGACTTCGGTGCGTATCTGGATGCGCTGGAAGCCGAGGTTGCACGAGGCGGTCTTATTGTGTTCCACAA                                                                                                                              <  1:139712/301‑1`  
                                                                                                                                                                                             `gCCAACGAACTGGCTGACAGTGCATCCACTAAAGTTACCGAGGCTGCCCGTGTGGCAAACCAAGCTCAACAGCTTTCCAAATTCTTTGAGTAATCAAACA‑GGAGAAACCATTATGATCGTTTCTGACATCGAAGCTAACGCCCTCTTAGAGAGCGTCACTAAGCTCCACTGCGGGGTTATCTACGACTACTCCACCGCTGAGTACGTAAGCTACCGTCCGAGTGACTTCGGTGCGTATCTGGATGCGCTGGAAGCCGAGGTTGCACGAGGCGGTCTTATTGTGTTCCACAACGGTCACAAG                                                                                                                    <  1:230792/300‑1`  
                                                                                                                                                                                                  `tataagagacagCAGTGCATCCACTAAAGTTACCGAGGCTGCCCGTGTGGCAAACCAAGCTCAACAGCTTTCCAAATTCTTTGAGTAATCAAACA‑GGAGAAACCATTATGATCGTTTCTGACATCGAAGCTAACGCCCTCTTAGAGAGCGTCACTAAGTTCCACTGCGGGGTTATCTACGACTACTCCACCGCTGAGTACGTAAGCTACCGTCCGAGTGACTTCGGTGCGTATCTGGATGCGCTGGAAGCCGAGGTTGCACGAGGCGGTCTTATTGTGTTCCACAACGGTCACAAGTATGA                                                                                                               <  1:130909/289‑1`  
                                                                                                                                                                                                  `CGAACTGTCTGACAGTTCATCCACTAAAGTTACCGAGGCTGCCCGTTTGTCAAACCAAGCTCAACAGCTTTCCAAATTCTTTGAGTAATCAAACA‑GGAGAAACCATTATGATCGTTTCTGACATCGAATCTAACGCCATCTTAGAGAGCGTCACTAAGTTCCACTGCGGGGTTATCTACGACTACTCCACCGCTGAGTACGTAAGCTACCGTCCGAGTGACTTCGGTGCGTATCTGGATGCGCTGGAAGCCGAGGTTGCACGAGGCGGTCTTATTGTGTTCCACAACGGTCACAAGTATGA                                                                                                               <  1:255849/301‑1`  
                                                                                                                                                                                                   `aAACTGGCTGACAGTGCATCCACTAAAGTTACCGAGGCTGCCCGTGTGGCAAACCAAGCTCAACAGCTTTCCAAATACTTGGAGTAATCAAACA‑GGAGAAAGCAATATGCTCGTTTTTGACATCGAAGCTAACGCCCTCTTAGATAGCGTCACTAAGTCCCACTGCGGGGTTATCTACGACTACTCCACCGCTGAGTACGTAATCTACCGTCCGAGTGACTTCGATGCGTATCTGGATGCGCTGGAAGCCGAGGTTGCACGAGGCGGTCTTATTGTGTTCCACAACGGTCACAAGTATGAC                                                                                                              <  1:95322/300‑1`  
                                                                                                                                                                                                    `acagggaaGACAGGGCATCCAGTAAAGATACCGAGGATGACCGTGTGGCAAACAAAGCTCAACAGCATTCCAAAATCATAGAGTAATCAAACA‑AGAGAAAACAGTATGATCGTTTCTGACATCGAAGCTAACGCCCTCTTAGAGAGCGTCACTAAGTTCCACTGCGGGGTTATCTACGACTACGCCACCGCTGAGTACGTAAGCTACCGTCCGAGTGACTTCGGTGCGTATCTGGATGCACTGGAAGCCGAGGTTGCACGAGGCGGTCTTATTGTGTTCCACAACGGTCACAAGTATGACa                                                                                                             <  1:457494/293‑2`  
                                                                                                                                                                                                    `aaagGGCTGACAGTGCATCCGCTAAAGTTACCGAGGCTGCCCGTGTGGCAAACCAAGCTCAACAGCTTTCCAAAGTCTTTGAGTAATCAAACA‑GGAGAAACCATTATGATCGTGTCTGACATCGAAGCTAACGACCTCTTAGAGAGCGTCACTAAGTTCCACTGCGGGGTTATCTACGACTACTCCACCGCTGAGTACGTAAGCTACCGTCCGAGTGACTTCGGTGCGTATCTGGATGCGCTGGAAGCCGAGGTTGCACGATGCGGTCTTATTGTGTTCCACAACGGGCACAAGTATGACG                                                                                                             <  1:233684/297‑1`  
                                                                                                                                                                                                     `ACTGGCTGACAGTGCAACCACTAAAGTTACCGAGGCTGCCCGTGTGGCAAACCAAGCTCAACAGCTTTCCAAATTCTTTGAGTAATCAAACA‑GGAGAAACCATTATGATCGTTTCTGACATCGAAGCTAACGCCCTCTTAGAGAGCGTCACTAAGTTCCACTGCGGGGTTATCTACGACTACTCCACCGCTGAGTACGTAAGCTACCGTCCGAGTTACTTCGGTGCGTATCTGGATGCGCAGGAAGCCGAGGTTGCACGAGGCGGTCTTATTGTGTTCCACAACGGTCACAAGTATGACGT                                                                                                            <  1:465140/301‑1`  
                                                                                                                                                                                                          `caGACAGGGCATCCACTAAAGTTACCGAGGCTGCCCGTGTGGCAAACCAAGCTCAACAGCTGTCCAAATTCTTTGAGTAATCAAACA‑GGAGAAACCATTATGATCGTTTCTGACATCGAAGCTAACGCCCTCTTAGAGAGCGTCACTAAGTTCCACTGCGGGGTTATCTACGACTACTCAACCGCTGAGTACGTAAGCTACCGTCCGAGTGACTTCGGTGCGTATCTGGATGCGCTGGAAGCCGAGGTTGCACGAGGCGGTCTTATTTTGTTCCACAACGGTCACAAGTATGACGTTCCTG                                                                                                       <  1:303025/299‑1`  
                                                                                                                                                                                                             `gCAGTGCATCCACTAAAGTTACCGAGGCTGCCCGTGTGGCAAACCAAGCTCAACAGCTTTCCAAATGCTTTGAGTAATCAAACA‑GGAGAAACCATTATGATCGTTTCTGACATCGAAGCTAACGCCCTCTTCGAGAGCGTCACTAAGTTCCACTGCGGGGTTATCTACGACTACTCCACCGCTGAGTACGTAAGCTACCGTCCGAGTGACTTCGGTGCGTATCTGGATGCGCTGGAAGCCGTGGTTGCACGAGGCGGTCTTATTGTGTTCCACAACGGTCACAAGTATGACGTTCCTGCAT                                                                                                    <  1:284315/300‑1`  
                                                                                                                                                                                                             `cagGTGCATCCACTAAAGTTACCGAGGCTGCCCGTGTGGCAAACCAAGCTCAACAGCTTTCCAAATTCTTTGAGTAATCAAACA‑GGAGAAACCATTATGATCGTTTCTGACATCGAAGTTAACGCCCTCTTAGAGAGCGTCACTAAGTTCCACTGCTGGGTTATCTACGACTACTCCACCGCTGAGTACGTAAGCTACCGTCCGAGTGACTTCGGTGCGTATCTGGATGCGCTGGAAGCCGAGGTTGCACGAGTCGGTCTTATTGTGTTCCACAACGGTCACAAGTATGACGTTCCTGCAT                                                                                                    <  1:190587/298‑1`  
                                                                                                                                                                                                             `aaAGTGCATCCACTAAAGTTACCGAGGCTGACCGAGTGGCAAACCAAGCTCAACAGCTTTCAAAATTCATTGAGTAATCAAACA‑GGTGAAACCATGATGATCGTTTCTGACATCGAAGCTAACGCCCTCTTAGAGGGCGTCACCAAGTTCCACTGCGGGGTTATCTACGAGTACTCCACAGCTGAGTACGTAAGCTACCGTCCGAGTGACGTCGGTGCGTATCTGGATGCGCTGGAAGCCGAGGTTGCACGAGGCGGTCTTATTGTGTTCCACAACGGTCACAAGTATGACGTTCCTGCAT                                                                                                    <  1:338517/299‑1`  
                                                                                                                                                                                                             `ACAGTGCATCCACTAAAGTTACCGAGGCTGCCCGTGTGGCAAACCAAGCTCAACAGCTTTCCAAATTCTTTGAGTAATCAAACA‑GGAGAAACCATTATGATCGTTTCTGACATCGAAGCTAACGCCCTCTTAGAGAGCGTCACTAAGTTCCACTGCGGGGTTATCTACGACTACTCCACCGCTGAGTACGTAAGCTACCGTCCGAGTGACTTCGGTGCGTATCTGGATGCGCTGGAAGCCGAGGTTGCACGAGGCGGTCTTATTGTGTTCCACAACGGTCACAAGTATGACGTTCCTGCAT                                                                                                    <  1:67028/301‑1`  
                                                                                                                                                                                                              `CAGTGCATCCACTAAAGTTACCGAGGCTGCCCGTGTGACAAACCAAGCTCAACAGCTTTCCAAATTCTTTGAGTAATCAAACA‑GGAGAAACCATTATGATCGTTTCTGACATCGCAGCTAACGCCCTCTTAGAGAGCGGCACTAAGTTCCACTGCGGGGTTATCTACGACTACTCCACCGCTGAGTACGTAAGCTACCGTCCGAGTGACTTCGGTGCGTATCTGGATGCGCTGGAAGCCGCGGTTGCACGAGGCCGTCTTATTGTTTTCCCCAACGGTCGCAAGTATGACGTTCCTTCATT                                                                                                   >  1:101400/1‑301`  
                                                                                                                                                                                                                       `CACTAAAGTTACCGAGGCTGCCCGTGTGGCAAACCAAGCTCAACAGCTTTCCAAATTCTTTGAGTAATC‑AATA‑GGAGAAATCAATATGATCGTTTCTGACATCGAAGCTAACGCCCTCTTAGAGAGCGTCACTAAGTTCCACTGCGGGGTTATCTACGACTACTCCACCGCTGAGTACGTAAGCTACCGTCCGAGTGACTTCGGTGCGTACCTGGATGCGCTGGAAGCCGAGGTTGCCCGAGGCGGTCTTATTGTGTTCCACAACGGTCACAAGTATGACGTTCCTGCATTGACCctgtct                                                                                         >  1:152117/1‑295`  
                                                                                                                                                                                                                         `CTAAAGTTACCGAGGCTGCCCGTGTGGCAAACCAAGCTCAACAGCTTTCCAAATTCTTTGAGTAATCAAACA‑GGAGAAACCATTATGATCGTTTCTGACATCGAAGCTAACGCCCTCTTAGAGAGCGTCACTAAGTTCCACTGCGGGGTTATCTACGACTACTCCACCGCTGAGGACGTAAGCTACCGTCCGAGTGACTTCGGTGCGTCTCTGGATGAGCTGGAAGCCGAGGTTGCACGAGGCGGTCTTATTGTGATCCACAACGGTCCCAAGTATGACGTTCCTGCACTGtctcttataa                                                                                        >  1:259892/1‑291`  
                                                                                                                                                                                                                              `GTTACCGAGGCTGCCCGTGTGGCAAACCAAGCTCAACAGCTTTCCAAATTCTTTGAGTAATC‑AATA‑GGAGAAATCAATATGATAGTTTCTGACATCGAAGCTAACGCCCTCTTAGAGAGCGTCACTAAGTTCCACTGCGGGGTTATCTACGACTACTCCACCGCTGAGTACGTAAGCTACCGTCCGAGTGACTTCGGTGCGTATCTGGATGCGCTGGAAGCCGAGGTTGCACGAGGCGGTCTTATTGTGTTCCACAACGGTCACAAGTATGACGTctgtctcttatcaccatctcctcacc                                                                                  >  1:116503/1‑275`  
                                                                                                                                                                                                                                      `gtgtataagagacagAAACCAAGCTCAACAGCTTTCCAAATTCTTTGAGTAATCAAACA‑GGAGAAACCATTATGATCGTTTCTGACATCGAAGCTAACGCCCTCTTAGAGAGCGTCACTAAGTTCCACTGCGGGGTTATCTACGACTACTCCACCGCTGAGTACGTAAGCTACCGTCCGAGTGACTTCGGTGCGTATCTGGATGCGCTGGAAGCCGAGGTTGCACGAGGCGGTCTTATTGTGTTCCACAACGGTCACAAGTATGACGTTCCTGCATTGACCAAACTGGCAAAGTTGCAATT                                                                           <  1:424354/286‑1`  
                                                                                                                                                                                                                                        `CTGCCCGTGTGGCAAACCAAGCTCAACAGCTTTCCAAATTCTTTGAGTAATCAAACA‑GGAGAAACCATTATGATCGTTTCTGACATCGAAGCTAACGCCCTCTTAGAGAGCGTCACTAAGTTCCACTGCGGGGTTATCTACGACTACTCCACCGCTGAGTACGTAAGCTACCGTCCGAGTGACCTCGGTGCGTATCTGGATGCGCTGGAAGCCGAGGTTGCACGAGGCGGTCTTATTGTGTTCCACAACGGTCACAAGTATGACGTTCCTGCATTGACCAAACTGGCAAAGTTGCAATTGA                                                                         >  1:320796/1‑301`  
                                                                                                                                                                                                                                                      `AACAAAGCTAAACAGCTTTCCAAATTCGTTGAGTAATCAAACA‑GGAGAAACCATTATGATCGTTTCTGACATCGAAGATAACGCCATATGAGAGAGCGTCACTAAGTTCCACTGCGGGGTTAGCTACGACTACTCCACCGCTGAGTACGTAATCTACCGTCCGAGTGACTTCGGTGCGTATCTGGATGCGCTGGAAGCCGAGGTTGCACGAGGCGGTCTTATTGTGTTCCACAACGGTCAAAAGTATGACGTTCCTGCATTGACCAAACTGGCAAAGTTGCAATTGAACCGAGAGTTCCAC                                                           <  1:40050/301‑1`  
                                                                                                                                                                                                                                                        `CCAAGCTCAACAGCTTTCCAAATTCTTTGAGTAATCAAACA‑GGAGAAACCATTATGCTCGTTTCTGACATCGAAGCTAACGCCCTCTTAGAGAGCGTCACTAAGTTCCACTGCGGGGTTATCTACGACTACTCCACCGCTGAGTACGTAAGCTACCGTCCGAGTGACTTCGGTGCGTATCTGGATGCGCTGGAAGCCGAGGTTGCACGAGGCGGTCTTATTGTGTTCCACAACGGTCACAAGTATGACGTTCCTGCATTGACCAAACTGGCAAAGTTGCAATTGAACAGAGAGTTCCACCT                                                         <  1:179487/301‑1`  
                                                                                                                                                                                                                                                          `AAGCTCACTATCTTTCCAAATTCTTTGAGTAATCACACA‑GTAGAAATCAATATGATCGTTTCTGACATCGAAGCTAACGCCCTCTTAGAGAGCGTCACTAAGTTCCACTGCGTGGTTATCTACGACTACTCCACCGCTGAGTACGTAAGCTACCGTCCGAGTGACTTCGGTGCGTATCTGGATTCGCTGGAAGCCGAGGTTGCACGAGGCGGTCTTATTGTGTTCCACAACGGTCACAAGTATGACGTTCCTGCATTGACCAAACTGGCAAAGTTGCAATTGAACCGAGAGTTCCACCTTC                                                       <  1:126490/301‑1`  
                                                                                                                                                                                                                                                                          `CAAATTCTTTGAGTAATCAAACA‑GGAGAAATCAATATGATCGTTTCTGACATCGAAGCTAACGCCCTCTTAGAGAGCGTCACTAAGTTCCACTGCGGGGTTATCTACGACTACTCCACCGCTGAGTACGTAAGCTACCGTCCGAGTGACTTCGGTGCGTATCTGGATGCGCTGGAAGCCGAGGTTGCACGAGGCGGTCTTATTGTGTTCCACAACGGTCACAAGTATGACGTTCCTGCATTGACCAAACTGGCAAAGTTGCAATTGAACCGAGAGTTCCACCTTCCTCGTGAGAACTGTAT                                       <  1:377052/301‑1`  
                                                                                                                                                                                                                                                                               `gagacagGTAATCAAACA‑GGAGAAACCATTATGATCGTTTCTGACATCGAAGCTAACGCCCTCTTAGAGATCGTCACTAAGTTCCACTGCGGGGTTATCTACGACTACTCCACCGCTGAGTACGTAAGCTACCGTCCGAGTGACTTCGGTGCGTATCTGGATGCGCTGGAAGCCGAGGTTGCACGAGGCGGTCTTATTGTGTTCCACAACGGTCACAAGTATGACGTTCCTGCATTGACCAAACTGGCAAAGTTGCAATTGAACCGAGAGTTCCACCTTCCTCGTGAGAACTGTATTGACA                                  <  1:414127/294‑1`  
                                                                                                                                                                                                                                                                                `CTTTGAGTAATCAAACA‑GGAGAAACCATTATGATCGTTTCTGACATCGAAGCTAACGCCCTCTTAGAGAGCGTCACTAAGTTCCACTGCGGGGTTATCTACGACTACTCCACCGCTGAGTACGTAAGCTACCGTCCGAGTGACTTCGGTGCGTATCTGGATGCGCTGGAAGCCGAGGTTGCACGAGGCGGTCTTATTGTGTTCCACAACGGTCACAAGTATGACGTTCCTGCATTGACCAAACTGGCAAAGTTGCAATTGAACCGAGAGTTCCACCTTCCTCGTGAGAACTGTATTGACAC                                 <  1:405361/301‑1`  
                                                                                                                                                                                                                                                                                        `AATCAAACA‑GGAGAAATCAATAGGATCGTTTCTGACATTGAAGCTAACGCCCTCTTAGAGAGCGTCATTAAGTTCCACTGCGGGGTTATCTACGACTACGCCACCGCTGAGTACGTAAGCTACCGTCCGAGTGACTTCGGTGCGTATCTGGATGCGCTGGAAGCCGAGGTTGCACGATGCGGTCTTATTGTGTTCCACAACGGTCACAAGTATGACGTTCCTGCATTGACCAAACTGGCAAAGTTGCAATTGAACCGAGAGTTCCACCTTCCTCGTGAGAACTGTATTGACACCCTTGTGT                         <  1:106238/301‑1`  
                                                                                                                                                                                                                                                                                        `AATCAAACA‑GGAGAAACCATTATGATCATTTCTGACATCGAAGCTAACGCCCTCTTAGAGAGCGTCACTAAGTTCCACTGCGGGGTTATCTACGACTACTCCACCGCTGAGTACGTAAGCTACCGTCCGAGTGACTTCGGTGCGTATCTGGATGCGCTGGAAGCCGAGGTTGCACGAGGCGGTCTTATTGTGTTCCACAACGGTCACAAGTATGACGTTCCTGCATTGACCAAACTGGCAAAGTTGCAATTGAACCGAGAGTTCCACCTTCCTCGTGAGAACTGTATTGACACCCTTGTGT                         <  1:486428/301‑1`  
                                                                                                                                                                                                                                                                                             `tataagagacagCCATTATGATCGTTTCTGACATCGAAGCTAACGCCCTCTTAGAGATCGTCACTAAGTTCCACGGCGGGGTTATCTACGACTACTCCACCGCTGAGTACGTAAGCTACCGTCCGAGTGACTTCGGTGCGTATCTGGATGCGCTGGAAGCCGAGGTTGCACGAGGCGGTCTTATTGTGTTCCACAACGGTCACAAGTATGACGTTCCTGCATTGACCAAACTGGCAAAGTTGCAATTGAACCGAGAGTTCCACCTTCCTCGTGAGAACTGTATTGACACCCTTGTGTTGTC                     <  1:352260/289‑1`  
                                                                                                                                                                                                                                                                                                         `CCATTATGATCGTTTCTGACATCGAAGCTAACGCCCTCTTAGAGAGCGTCACTAAGTTCCACTGCGGGGTTATCTACGACTACTCAACCGCTGAGTACGTAAGCTACCGTCCGAGTGACTTCGGTGCGTATCTGGATGCGCTGGAAGCCGAGGTTGCACGAGGCGGTCTTATTGTGTTCCACAACGGTCACAAGTCTGACGTTCCTGCATTGACCAAACTGGCAAAGTTGCAATTGAACCGAGAGTTCCACCTTCCTCGTGAGAACTGTATTGACACCCTTGTGTTGTCACGTTTGATTac         >  1:77719/1‑299`  
                                                                                                                                                                                                                                                                                                                `GATCGTTTATGAAATCGAAGCTAACGCCCTCTTAGAGAGCGTCACTAAGTTCCACTGCGGGGTTATCTACGACTACTCCACCGCTGAGTACGTAAGCTACCGTCCGAGTGACTTCGGTGCGTATCTGGATGCGCTGGAAGCCGAGGTTGCACGAGGCGGTCTTATTGTGTTCCACAACGGTCACAAGTATGACGTTCCTGCATTGACCAAACTGGCAAAGTTGCAATTGAACCGAGAGTTCCACCTTCCTCGTGAGAACTGTATTGACACCCTTGTGTTGTCACGTTTGATTCATTCCAAC  <  1:57291/301‑1`  

                                                                                                                                                                                                                                                                                                                                                                                                                                                                                                                                                                                                                               
`TTCCAGACGACTACGAGAAGTTTCGCTGGAGAGTCCCATTCTAATACGACTCACTAAAGGAGACACACCATGTTCAAACTGATTAAGAAGTTAGGCCAACTGCTGGTTCGTATGTACAACGTGGAAGCCAAGCGACTGAACGATGAGGCTCGTAAAGAGGCCACACAGTCACGCGCTCTGGCGATTCGCTCCAACGAACTGGCTGACAGTGCATCCACTAAAGTTACCGAGGCTGCCCGTGTGGCAAACCAAGCTCAACAGCTTTCCAAATTCTTTGAGTAATCAAACA‑GGAGAAACCATTATG‑‑‑‑‑‑‑‑‑‑‑‑‑‑‑‑‑‑‑‑‑‑‑‑‑‑‑‑‑‑‑‑‑‑‑‑‑‑‑‑‑‑‑‑‑‑‑‑‑‑‑‑‑‑‑‑‑‑‑‑‑‑‑‑‑‑‑‑‑‑‑‑‑‑‑‑‑‑‑‑‑‑‑‑‑‑‑‑‑‑‑‑‑‑‑‑‑‑‑‑‑‑‑‑‑‑‑‑‑‑‑‑‑‑‑‑‑‑‑‑‑‑‑‑‑‑‑‑‑‑‑‑‑‑‑‑‑‑‑‑‑‑‑‑‑‑‑‑‑‑‑‑‑‑‑‑‑‑‑‑‑‑‑‑‑‑‑‑‑‑‑‑‑‑‑‑‑‑‑‑‑‑‑‑‑‑‑‑‑‑‑‑‑‑‑‑‑‑‑‑‑‑‑‑‑‑‑‑‑‑‑‑‑‑‑‑‑‑‑‑‑‑‑‑‑‑‑‑‑‑‑‑‑‑‑‑‑‑‑‑‑‑‑‑‑‑‑‑‑‑‑‑‑‑‑‑‑‑‑‑‑‑‑‑‑‑‑‑‑‑‑‑‑‑‑‑‑‑‑‑‑‑‑‑‑‑‑‑‑‑‑‑‑‑‑‑‑‑‑‑  >  NC_001604.1/13283‑13586`  
`‑‑‑‑‑‑‑‑‑‑‑‑‑‑‑‑‑‑‑‑‑‑‑‑‑‑‑‑‑‑‑‑‑‑‑‑‑‑‑‑‑‑‑‑‑‑‑‑‑‑‑‑‑‑‑‑‑‑‑‑‑‑‑‑‑‑‑‑‑‑‑‑‑‑‑‑‑‑‑‑‑‑‑‑‑‑‑‑‑‑‑‑‑‑‑‑‑‑‑‑‑‑‑‑‑‑‑‑‑‑‑‑‑‑‑‑‑‑‑‑‑‑‑‑‑‑‑‑‑‑‑‑‑‑‑‑‑‑‑‑‑‑‑‑‑‑‑‑‑‑‑‑‑‑‑‑‑‑‑‑‑‑‑‑‑‑‑‑‑‑‑‑‑‑‑‑‑‑‑‑‑‑‑‑‑‑‑‑‑‑‑‑‑‑‑‑‑‑‑‑‑‑‑‑‑‑‑‑‑‑‑‑‑‑‑‑‑‑‑‑‑‑‑‑‑‑‑‑‑‑‑‑‑‑‑‑‑‑‑‑‑‑‑‑‑‑‑‑‑‑‑‑‑‑‑‑‑‑‑‑‑‑‑‑‑‑‑‑‑‑‑‑‑‑‑‑‑‑‑‑‑‑‑‑‑‑‑‑‑‑‑‑‑‑‑‑‑‑‑‑‑tatgATCGTTTCTGACATCGAAGCTAACGCCCTCTTAGAGAGCGTCACTAAGTTCCACTGCGGGGTTATCTACGACTACTCCACCGCTGAGTACGTAAGCTACCGTCCGAGTGACTTCGGTGCGTATCTGGATGCGCTGGAAGCCGAGGTTGCACGAGGCGGTCTTATTGTGTTCCACAACGGTCACAAGTATGACGTTCCTGCATTGACCAAACTGGCAAAGTTGCAATTGAACCGAGAGTTCCACCTTCCTCGTGAGAACTGTATTGACACCCTTGTGTTGTCACGTTTGATTCATTCCAAC  >  NC_001604.1/14356‑14655`

  

Alignment Legend

Aligned base mismatch/match (shaded by quality score): `ATCG/ATCG` `< 3 ≤` `ATCG/ATCG` `< 9 ≤` `ATCG/ATCG` `< 18 ≤` `ATCG/ATCG` `< 32 ≤` `ATCG/ATCG` `< 38 ≤` `ATCG/ATCG`

Unaligned base: `atcg`    Masked matching base: `atcg`    Alignment gap: `‑`    Deleted base: `‑`

Reads not counted as support for junction

read\_name Not counted due to insufficient overlap past the breakpoint.

read\_name Not counted due to not crossing MOB target site duplication.
